# Older Adults’ Motivation and Volition in Sustaining Exercise After a Structured Program

**DOI:** 10.1093/geroni/igaf122.3985

**Published:** 2025-12-31

**Authors:** Bora Jin, Elizabeth Roumell

**Affiliations:** Winston Salem State University, Kernersville, North Carolina, United States; Texas A&M University, Texas A&M University, Texas, United States

## Abstract

Sustaining exercise habits is important for supporting health and independence in later life (World Health Organization, 2024). While exercise adherence has been widely studied, less is known about how older adults continue activity once structured programs conclude. Drawing on Self-Determination Theory (Ryan & Deci, 2017) and Volitional Competences (Kuhl, 2000; Elsborg et al., 2019), we explored how motivation and volition influence exercise continuation among older adults who completed an eight-week structured exercise program. Using a critical realist approach (Fletcher, 2017), we conducted 29 semi-structured interviews, analyzed through abductive and retroductive reasoning, and coded with Atlas.ti. We identified motivational factors that explained why participants continued exercising (e.g., perceived physical improvements, accountability, intergenerational influence, and social connection and enjoyment). We also found volitional factors that described how participants sustained routines despite challenges, including embedding exercise into daily life, using self-regulation strategies, adapting routines to prevent injury, and drawing on a lifelong exerciser identity. Participants also described barriers such as health limitations, emotional and cognitive challenges, hesitancy to act once programs ended, and financial or logistical constraints. Our findings suggest that older adults build on motivation to engage and volition to persist once a structured program ends, balancing supportive daily practices with barriers such as health concerns, emotional challenges, and financial constraints. We consider how autonomy-supportive practices, accountability structures, and program designs that acknowledge these barriers may help sustain participation. We conclude by outlining implications for fitness instructors, health educators, and practitioners seeking to promote meaningful exercise engagement in older adults.

